# Differing Serum Cea in Primary and Recurrent Rectal Cancer - A Reflection of Histology?

**DOI:** 10.4021/wjon479w

**Published:** 2012-04-23

**Authors:** Angela C Chang, Leigh R Warren, Savio G Barreto, Randolph Williams

**Affiliations:** a Department of Surgery, Modbury Hospital, South Australia, Australia; b These authors contributed equally to this manuscript

**Keywords:** Cancer, CEA, Colorectal, Rectal

## Abstract

**Background:**

Serum carcinoembryonic antigen (CEA) levels are not universally measured in colorectal cancers. CEA levels have been reported to be usually normal at time of primary rectal cancer diagnosis but elevated in recurrent disease. The aims of the study were to (1) compare serum CEA levels performed at time of primary and recurrent colorectal tumour diagnosis; and (2) to determine serum CEA levels in rectal cancers at primary diagnosis to analyse potential factors influencing differing CEA levels.

**Methods:**

A retrospective analysis of patients treated for colorectal cancers at Modbury Hospital, South Australia was performed. Each admission was reviewed within the electronic database. Serum CEA levels and tumour-related factors were determined in patients who underwent curative surgery for their primary tumour and developed tumour recurrence/metastases within the study period.

**Results:**

438 patients were treated for colorectal cancer in the study period. In patients who underwent curative surgery and developed a recurrence, serum CEA was elevated in 20% patients at primary diagnosis and in 46.6% patients at recurrence. Only 1 of 30 patients with rectal cancer had an elevated CEA at diagnosis of primary tumour. Tumour relationship to the peritoneal reflection did not appear to play a role.

**Conclusions:**

In rectal cancers, serum CEA levels are often normal at the time of initial diagnosis. However, this should not preclude its use in post-operative surveillance. Serum CEA levels noted in primary rectal cancer appear unrelated to the relationship of the tumour to the peritoneal reflection. Stroma-related factors could possibly be involved and merit further investigation.

## Introduction

Carcinoembryonic antigen (CEA) is a tumour marker widely measured in colorectal cancer (CRC) [1] at time of primary cancer diagnosis and as part of the intensive surveillance [2-4]. This has led to improved outcomes [2, 3, 5]. Although recommended in many countries [1, 6], Australia’s previous National Health and Medical Research Council (NHMRC) guidelines [7] did not favour routine measurement of CEA for all CRC patients. The current guidelines do recommend its performance [8]. The presence of a normal preoperative serum CEA has confounded the use of CEA in follow-up in other countries [9].

Patients with rectal tumours are less likely to present with a raised serum CEA level. Grossmann et al [10] observed that in patients with primary rectal cancer and a normal CEA, CEA became elevated in up to 50% on follow-up in the presence of recurrent disease. The authors did not provide a clear hypothesis for this observation.

The aims of the current study were to 1) compare the serum CEA levels at time of primary and recurrent CRC diagnosis and 2) determine serum CEA levels in rectal cancers at primary diagnosis, as per Grossmann et al [10], and to identify potential factors influencing differing CEA levels.

## Material and Methods

A retrospective search of a prospectively maintained electronic database of a public teaching hospital was undertaken. International Classification of Diseases (ICD) codes for colon and rectum cancers for a 58-month period, from January 2007 to October 2011 were analysed with the aim of identifying all patients treated for colorectal cancer at the hospital. The overall cohort included patients who: a) Underwent surgery - for primary tumour or recurrence; b) Underwent chemotherapy - adjuvant or palliative, or; c) Received palliative care - for metastatic disease or local recurrence, at the hospital in the study period.

Each admission was reviewed within the electronic database. The case records of patients identified to have a primary tumour and a tumour recurrence within the stated study period were manually reviewed.

### Variables collected for each patient

Age, sex, serum CEA level (µg/L) at diagnosis of primary tumour and/or at time of recurrence, surgery, tumour histology, and primary and metastatic tumour location. Each tumour was additionally restaged based on the revised American Joint Committee on Cancer (AJCC) staging and the International Union against Cancer (UICC) TNM staging [11].

### Inclusion criteria for evaluation of CEA in recurrent disease

Patients who underwent curative surgery for their primary tumour and developed tumour recurrence/metastases within the study period.

### Inclusion criteria for evaluation of CEA in primary rectal cancer and definitions

Patients with primary, non-metastatic (distant) rectal cancer within the study period. Lymph node involvement on histology was not considered metastatic.

For the analysis of CEA levels in primary rectal cancer, the peritoneal reflection [12] was regarded as the landmark dividing the high and the low rectum based on the biologic barrier between lymphatic pathways. Rectal adenocarcinomas above the reflection were classified as ‘high’ while rectal and anal canal adenocarcinomas below the reflection were classified as ‘low’. Anal canal adenocarcinomas were grouped with rectal cancers owing to a previous study indicating a similar CEA expression profile between these two sites [13]. For the purpose of this study, ‘recurrent’ cancer refers to local recurrence and distal metastases.

This was a retrospective study. No experiments we conducted on human or animal subjects. Identifying patient details were available to the study investigators only.

### Statistical analysis

The data was compiled and analysed using Microsoft Excel 2007 (Microsoft, Redmond, California). Where applicable, data has been provided as median (range).

## Results

A total of 438 patients were treated for different stages of colorectal cancers at the hospital during the study period. These included 233 male patients (53.2%) and 205 female patients (46.8%). The median age was 72 years (range: 21 - 102).

### CEA in Recurrent disease

Of the 438 patients, 46 patients (10.5%) underwent curative surgery for a primary tumour and developed tumour recurrence/metastases within the study period thereby fulfilling the criteria for inclusion. Of these, only 15 patients had CEA levels measured at the time of primary and recurrent cancer diagnosis. The data of these patients has been summarised in Table 1. CEA was elevated in only 3 patients (20%) with primary colorectal cancers and in 7 patients (46.6%) with recurrent or metastatic cancers.

**Table 1:** Data of the 15 Patients With CEA Levels Available at the Time of Primary Tumour and Recurrence

Patient Demographics			Primary Crc		Recurrent Crcv	
Gender	Age	CEA	Location	Stage	CEA	Location
**CEA normal primary CRC → CEA normal recurrent colorectal carcinoma**						
Male	64	2.3	High rectum	3b	7	Liver
Male	72	1	Sigmoid	2a	1	Liver
Female	56	8	High rectum	2b	2	Pelvic wall
Male	84	2	Transverse colon	3b	4	Brain, lungs
Male	78	2	Descending colon	3c	1	Liver, spleen
Male	70	3.4	Caecum	3b	1	Brain
Female	77	3	Sigmoid	3c	2	Liver
**CEA normal primary CRC → CEA elevated recurrent colorectal carcinoma**						
Male	64	2.3	Low rectum	3b	98	Lung
Female	80	1.6	Caecum	2b	6.3*	Liver
Male	69	1	Low rectum	2a	52	Local
Male	80	1	Low rectum	2a	17	Liver
**CEA elevated primary CRC → CEA elevated recurrent colorectal carcinoma**						
Female	72	39.3	Rectosigmoid	3b	180.9	Liver
Male	73	15	Rectosigmoid	3b	11	Liver, lung
Male	73	211.8	Transverse colon	3c	6975	Lung
**CEA elevated primary CRC → CEA normal recurrent colorectal carcinoma**						
Female	60	14.4	Caecum	3b	1	Small bowel

* The normal range for CEA was defined as < 5 µg/L by the laboratory in which this patient underwent her test as opposed to < 10 µg/L in the other patients.

### CEA in rectal cancer

Of the overall colorectal cancer cohort of 438 patients, 79 patients were treated for rectal cancers. This included 50 male patients (63%) and 29 female patients (36.7%) with a median age of 67 years (range: 39 - 102).

Of the 79 patients, relevant data on the CEA levels was available in 39 patients (49.3%). Of the 39 patients, 30 patients had primary, non-metastatic (distant) rectal cancer and were included for further analysis.

Table 2 shows the rectal tumour location and CEA level of these patients.

**Table 2:** Data of the 30 Patients With CEA Levels Available at the Time of Primary, Non Metastatic Rectal Cancer Based on Tumour Relation to the Reflection of the Peritoneal Fold

Tumour location	CEA normal	CEA elevated	Total patients
High	12	1	13
Low	17	0	17

CEA: Carcinoembryonic antigen.

## Discussion

The data from our study indicates that CEA is of more value in detecting recurrent rather than primary colorectal cancers amongst the cohort of patients who underwent primary curative surgery and developed a recurrence, and in whom CEA levels were available for comparison at the two time points within the study period. There was no obvious correlation between the stage of primary disease and serum CEA levels in this data. The findings from this study were consistent with those of Grossmann et al [10] that while primary rectal cancers, unlike colon cancers, may not be accompanied by an elevated serum CEA level, CEA levels may be elevated in recurrent disease. This supports the routine use of serum CEA in follow-up protocols of patients with rectal cancers. Our data did not indicate a correlation between the anatomical relationship of the tumour to the peritoneal reflection.

The latter inference indicates that there must be other reasons for the differential expression of serum CEA in primary and recurrent rectal cancers. In 1993, Ng et al [14] noted a significant positive correlation between CEA imunoperoxidase staining in the basolateral regions of cells and stroma of colorectal cancers and elevated plasma CEA levels. We hypothesized that the difference in serum CEA levels between colon and rectal cancer may be related to differing factors in their stroma.

In order to explain the lack of elevation in serum CEA in primary rectal cancers, we thus sought to determine if there existed any such differences between the colonic and rectal tissue.

Multinucleated stromal giant cells are relatively more abundant in the colonic stroma [15] as opposed to the rectum. When involved in carcinomas of the breast, these cells have been shown to stain positively for CEA [16]. Possibly these cells are involved in the expression of serum CEA at other sites. These multinucleate giant cells express matrix metalloproteinase-9 (MMP-9) which is responsible for the accelerated breakdown of the extracellular matrix associated with tumour invasion and metastases [17].

Another potentially relevant difference in stroma between the colon and rectum lies in the distribution of myofibroblasts. Myofibroblasts have been shown to regulate a number of tumour-promoting functions, including angiogenesis, invasion and metastasis and are generally located at the invasive front of tumours [18]. More than 80% of the fibroblast-like cells located at the leading edge of colon cancer were found to be myofibroblasts [19]. This is in stark contrast to the rectal cancer where myofibroblasts are less extensively distributed in the more prevalent intermediate and mature fibrotic stroma.

CEA is an E- and L-selectin ligand and hence may play a role in metastasis [20]. It is possible that the differing stromal factors between colon and rectal cancers may account for some of the differences in serum CEA levels between cancers located in the two sections of the large bowel.

An elevation in CEA accompanying the recurrence or metastasis of rectal cancer in cases where the CEA was normal in the primary tumour can also be explained, at least in part, by the role of myofibroblasts. Recent data has provided evidence that in the case of metastases, for instance in the liver, myofibroblasts develop from hepatic stellate cells native to the metastatic site [21] and not from the primary tumour.

One of the limitations of our study is the small number of patients in the study cohort despite a large denominator in terms of the overall cohort. This suggests that serum CEA was not performed regularly for many of the patients in the initial cohort. While this could potentially reflect the older recommendations from the NHMRC in which performance of CEA was not considered mandatory, it could also simply indicate the resultant individual clinicians practice protocols.

### Conclusions

Measuring serum CEA is more useful in post-operative surveillance of colorectal cancer compared to performance at the time of primary tumour diagnosis. In rectal cancers, normal serum CEA levels are commonly encountered at the time of diagnosis of the primary tumour. However, this should not preclude its use in the post-operative surveillance. Serum CEA levels noted in primary rectal cancer appear unrelated to the relationship of the tumour to the peritoneal reflection, although, stroma-related factors could possibly be involved and merit further investigation.
